# Epidemiology and Time Series Analysis of Human Brucellosis in Tebessa Province, Algeria, from 2000 to 2020

**DOI:** 10.34172/jrhs.2022.79

**Published:** 2021-10-31

**Authors:** Seif Eddine Akermi, Mohamed L’Hadj, Schehrazad Selmane

**Affiliations:** ^1^L’IFORCE, Faculty of Mathematics, University of Sciences and Technology Houari Boumediene, Algiers, Algeria; ^2^Beni Messous University Hospital Centre, Ministry of Health, Population and Hospital Reform, Algiers, Algeria

**Keywords:** Human brucellosis, Neural network auto-regressive mode, Prediction, SARIMA model, Tebessa province

## Abstract

**Background:** Brucellosis runs rampant endemically with sporadic outbreaks in Algeria. The present study aimed to provide insights into the epidemiology of brucellosis and compare the performance of some prediction models using surveillance data from Tebessa province, Algeria.

**Study Design:** A retrospective study.

**Methods:** Seasonal autoregressive integrated moving average (SARIMA), neural network autoregressive (NNAR), and hybrid SARIMA-NNAR models were developed to predict monthly brucellosis notifications. The prediction performance of these models was compared using root mean square error (RMSE), mean absolute error (MAE), and mean absolute percentage error (MAPE).

**Results:** Overall, 13670 human brucellosis cases were notified in Tebessa province from 2000-2020 with a male-to-female ratio of 1.3. The most affected age group was 15-44 years (56.2%). The cases were reported throughout the year with manifest seasonality. The annual notification rate ranged from 30.9 (2013) to 246.7 (2005) per 100000 inhabitants. The disease was not evenly distributed, rather spatial and temporal variability was observed. The SARIMA (2,1,3) (1,1,1)_12^'^_, NNAR (12,1,6)_12^'^_, and SARIMA (2,0,2) (1,1,0)_12_- NNAR (5,1,4)_12_ were selected as the best-fitting models. The RMSE, MAE, and MAPE of the SARIMA and SARIMA-NNAR models were by far lower than those of the NNAR model. Moreover, the SARIMA-NNNAR hybrid model achieved a slightly better prediction accuracy for 2020 than the SARIMA model.

**Conclusion:** As evidenced by the obtained results, both SARIMA and hybrid SARIMA-NNAR models are suitable to predict human brucellosis cases with high accuracy. Reasonable predictions, along with mapping brucellosis incidence, could be of great help to veterinary and health policymakers in the development of informed, effective, and targeted policies, as well as timely interventions.

## Background

 Brucellosis, a zoonotic infection caused by the bacterial genus *Brucella,* features among the main five zoonotic diseases which the Department of Control of Neglected Tropical Diseases deals with.^1^ This disease is characterized by a large clinical polymorphism and not very specific manifestations, leading to serious complications often requiring hospitalization with long and restrictive treatments. The major pathways of contamination are the digestive tract through the ingestion of contaminated products (milk and its derivatives) and direct contact with infected animals.^1^ The mucocutaneous passageway of the bacteria in humans occurs following contact with infected animals, including abortion, calving, excreta, soiled litter, viscera, and carcasses. Contamination can also occur accidentally in laboratories or by inhalation of infected dust or aerosols.^1^

 Although brucellosis has been eradicated from several developed countries, it remains a major public health problem in numerous parts of the world, notably Africa, the Mediterranean basin, South America, and Asia. The prevalence of this disease varies considerably from country to country. Each year, approximately 500 000 incident cases of human brucellosis are reported; nevertheless, the actual incidence is estimated to be 10 to 25 times higher. Underreporting and absence of an effective program to monitor the disease in many endemic countries preclude an accurate overview of situation.^2-5^

 Classified second after leishmaniasis among zoonotic diseases in Algeria, brucellosis poses a serious challenge to the health of animals and humans, running rampant endemically with sporadic outbreaks and high variability across the provinces in the country. The most common species incriminated in human pathology are *Brucella melitensis* and *Brucella abortus*. The disease is notifiable and a national veterinary program to fight it has been in effect since 2009. Official data for animal brucellosis are still difficult to analyze, as long as the screening concerns only dairy cattle herds; moreover, they are only tracked down episodically.^6^ In 2010, 10 014 human cases had been reported, and this number decreased by almost half in 2011 due to good inter-sectoral collaboration. Nevertheless, a linear upward trend has been observed since 2014. The recorded cases have risen from 5533 cases in 2014 to 10 198 cases in 2017. The highland rearing areas remain the most active foci of the disease.^7^

 Tebessa, an endemo-epidemic province, leads the eastern part of the country in terms of notification cases, making it a suitable choice to provide an overview on the epidemiological features of human brucellosis, perform statistical analysis, examine the spatio-temporal distribution at the municipal level, and develop predictive models.

## Methods

###  Study region

 Tebessa province is located in the extreme east of Algeria. The region is an agro-pastoral area, 38% of which is agricultural land. The population was estimated at 794 528 inhabitants in 2020, 70% of whom live in rural areas.^8^

###  Data

 The daily diagnosed human brucellosis cases confirmed by the laboratory between 2000 and 2020, provided by the Tebessa Department of Public Health (TDPH), have been aggregated into monthly data. The data set has been divided into three subsets: a training set from January 2000 to December 2015 used for model identification, a test set from January 2016 to December 2019 used for forecasting accuracy, and data from January 2020 to December 2020 used for predictions. This study was approved by TDPH; the proposal was sent on January 2, 2020 and was approved on February 6, 2020.

###  Modeling methods

 Among popular statistical models used to model recorded cases over time and make predictions, we can refer to Autoregressive (AR) Moving Average (MA) models^9-11^which are used as a reference point for the evaluation of new prediction methods. Artificial neural networks (ANNs) and combined models have been developed to improve predictions.^12-14^

###  Seasonal autoregressive moving average model 

 Given the observed seasonal trend in the monthly data and following the recommendations of Box and Jenkins, the seasonal autoregressive moving average (SARIMA) model was considered in the first instance. In the sequel, we briefly review the Box and Jenkins methodology to build SARIMA model.^10,11,15^

 Given a stationary time series of data $X_{t}\left(t=1,...,n\right)$ the SARIMA model, denoted by $\left(p,d,q\right){\left(P,D,Q\right)}_{S}$ can be expressed by the following difference equation:


$$\phi_{p}\left(B\right)\Phi_{p}\left(B^{S}\right)\nabla^{d}\nabla_{S}^{D}X_{t}=\theta_{q}\left(B\right)\Theta_{Q}\left(B^{S}\right)\varepsilon_{t}.$$

Where the backward shift operator 𝐵 is defined as $B^{k}X_{t}=X_{t-k},S$ presenting the seasonality period, 𝑑 is the number of non-seasonal differences, 𝐷 is the degree of seasonal integration, 𝑝 is the number of AR terms, 𝑃 is the degree of seasonal AR terms, 𝑞 signifies by is the number of MA terms, 𝑄 is the degree of seasonal MA model, ∇ = 1−𝐵 is the differencing operator $\nabla_{s}=1-B^{s}$ , notes the seasonal differencing operator, and $\varepsilon_{t}$ is the white noise process. The polynomials $\phi_{p}\left(B\right),\Phi_{p}\left(B\right),\theta_{q}\left(B\right),\Theta_{Q}\left(B\right)$ are AR, MA, seasonal AR, and seasonal MA polynomials, respectively.

 The SARIMA modeling is best performed while following a protocol. The first step is to check the stationary condition. To this end, the augmented Dickey-Fuller unit-root (ADF) test was used.^16^ To stabilize the variance of a time series that exhibits non-stationary variance, such transformations as logarithm, square root, and reciprocal can be applied to each observation $X_{t}\left(t=1,...,n\right)$ To stabilize the mean, an appropriate order of differences can render a non-stationary series a stationary one. The process for the identification of order of the AR and MA operators is provided in.^10,11,15^ The model equation is thereafter estimated. To test the model for goodness-of-fit, the residuals are analyzed. The residuals should be uncorrelated with a mean of zero and follow a Gaussian distribution; moreover, the autocorrelations of the residuals should not be significantly different from zero. The correlation structure provides various choices for 𝑝 and 𝑞 values, thereby generating several models. The best-fit model selection is based on such criteria as the smallest Akaike information criterion (AIC), smallest root mean squared error (RMSE), and the highest adjusted R^2^ in addition to the stationary and invertibility condition, as well as the white noise condition for residuals.^11,15^

###  Neural network model

 Given the observed nonlinear trend in the data, ANN is among the appropriate models that can be used to approximate various nonlinearities in the data. The most widely used model for time series modeling and forecasting is the single hidden layer feed-forward network. This model is characterized by a network of three layers, namely input layer (input variables), hidden layer (layers of nodes between the input and output layers), and output layer (output variables) of simple processing units which are connected by acyclic links.

 The relationship between the output *y*_t_ and $y_{t-1},y_{t-2},...,y_{t-p}$ is formalized as follows:


$$y_{t}=\alpha_{o}+\sum_{j=1}^{q}\alpha_{j}g\left(\beta_{oj}+\sum_{i=1}^{p}\beta_{ij}y_{t-1}\right)+\varepsilon_{t}$$

where 𝑝 is the number of input nodes, 𝑞 is the number of hidden nodes, $\alpha_{j}\left(j=0,1,...,q\right)$ and $\beta_{ij}\left(i=0,1,...,p;j=1,...,q\right)$ are parameters of the model, and 𝑔 signifies the hidden layer transfer function. The logistic function as defined by


$$g\left(x\right)=\frac{1}{1+e^{-x}}$$

was used as the hidden layer transfer function. It is noteworthy that the neural network and non-linear AR model have similar representation.^13,14,17^

###  Hybrid SARIMA-NNAR forecasting

 Almost all real-world time series contain both linear and non-linear correlation structures among the observations. Neither autoregressive integrated moving average (ARIMA) nor ANN is universally suitable for all types of time series. Indeed, the approximation of nonlinear time series by ARIMA models or linear time series by ANN models may not be appropriate.^14^ In this study, we used $NNAR{\left(p,P,k\right)}_{m}$ model.^17^ The NNAR model is one type of ANN model, in which lagged values of data can be used as inputs to the neural network. The $NNAR{\left(p,P,k\right)}_{m}$ model has inputs $\left(y_{t-1},y_{t-2},...,y_{t-p},y_{t-m},y_{t-2m},...,y_{t-Pm}\right)$ and *k* neurons in the hidden layer. A $NNAR{\left(p,P,0\right)}_{m}$ model is equivalent to a $ARIMA\left(p,0,0\right){\left(P,,0,0\right)}_{m}$ model but without restrictions on the parameters that ensure stationarity. To combine the linear and nonlinear models, the hybrid methodology is proposed. To perform the hybrid methodology, the original time series at time 𝑡 needs to be composed of an auto-correlated linear (*L*_t_) and nonlinear (*N*_t_).^17^

 Firstly, the SARIMA model is used to capture the linear component in the data; thereafter, NNAR is used to capture the nonlinear component in the residuals part. The residuals are expressed as $e_{t}=y_{t}-{\hat{L}}_{t}$ where ${\hat{L}}_{t}$ is the forecasting value at time t of *y*_t_, estimated by SARIMA model and are represented as follows:


$$e_{t}=f\left(e_{t-1},e_{t-2},...,e_{t-p}\right)+\varepsilon_{t}={\hat{N}}_{t}+\varepsilon_{t}.$$

Where p is the optimal number of lags, $o_{t}$ is the white noise, ${\hat{N}}_{t}$ signifies the forecast value at time 𝑡 by NNAR model, and 𝑓 refers to a nonlinear function determined by the multilayer perceptron.^14^

 The linear and nonlinear forecasting values obtained by SARIMA and NNARs models are then combined to get the forecast:


$${\hat{z}}_{t}={\hat{L}}_{t}+{\hat{N}}_{t}.$$

###  Measures of accuracy

 The frequently used metrics to measure performance, estimate the accuracy of the forecasts, and compare different models are:

 Root mean square error $RMSE=\sqrt{\frac{1}{n}\sum_{t=1}^{n}{\left(X_{t}-{\hat{X}}_{t}\right)}^{2}}$ .

 Mean absolute error $MAE=\frac{1}{n}\sum_{t=1}^{n}\left|X_{t}-\left.{\hat{X}}_{t}\right|\right.$

 an absolute percentage error $MAPE=\frac{1}{n}\sum_{t=1}^{n}\frac{\left|X_{t}-{\hat{X}}_{t}\right|}{X_{t}}$

 here 𝑛 is the size of the test set, ${\hat{X}}_{t}$ signifies the forecasted observation, and $X_{t}$ denotes the actual observation at time 𝑡. A model with lowest value of the error measurements indicates a better performance model.^4,15^ Models were built using TSA package, forecast package, neural net package, and the nnetar function in the forecast package for R to fit a neural network model to a time series with lagged values of the time series as inputs and forecast Hybrid package under R software (version 3.4.4) (Network Theory Ltd., Bristol, UK).^17^

## Results

###  A statistical appraisal of human brucellosis in Tebessa province

**Figure 1:**
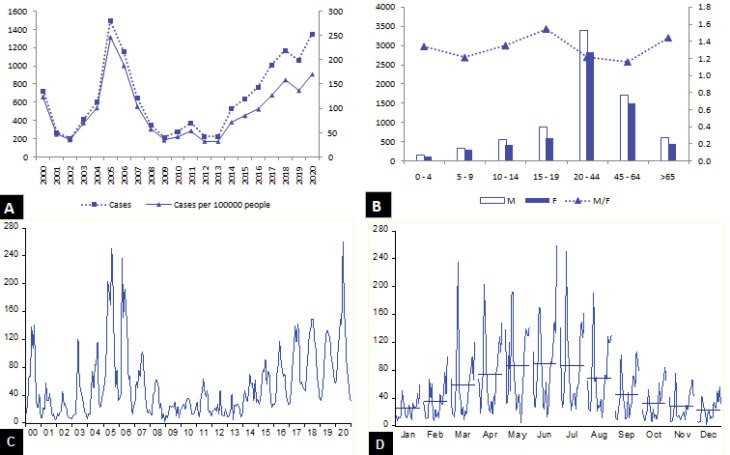
Yearly evolution (A), age group distribution (B), monthly (C), and the seasonal graph (D) of reported human Brucellosis cases in Tebessa province 2000-2020. Blue bars in Figure (D) represent the monthly average of cases.

 The annual evolution of human brucellosis cases in Tebessa province demonstrated that this disease is endemic and surges into outbreak states periodically (Figure 1A). Over the 21-year study period, 13 670 human brucellosis cases were notified, out of whom 7592 (55.5%) and 6058 (44.3%) cases were male and female, respectively, and gender was missing for 20 subjects. The mean score of yearly notifications was 651 ± 405.6 (95% CI: 466.32, 835.32). A peak in notifications was reached in 2005 (1495 cases), subsequently, a gradual decrease was observed until 2009, and a gradual increase was observed since 2013, reaching 1342 cases in 2020. Out of all reported cases, 13.1% were children under the age of 14 years, the 20-44 age group gathered 45.6% of notifications, followed by the age group of 45-64 years with 23.3% of cases (Figure 1B). It is noteworthy that 57% of the notified cases were from rural areas.

**Figure 2:**
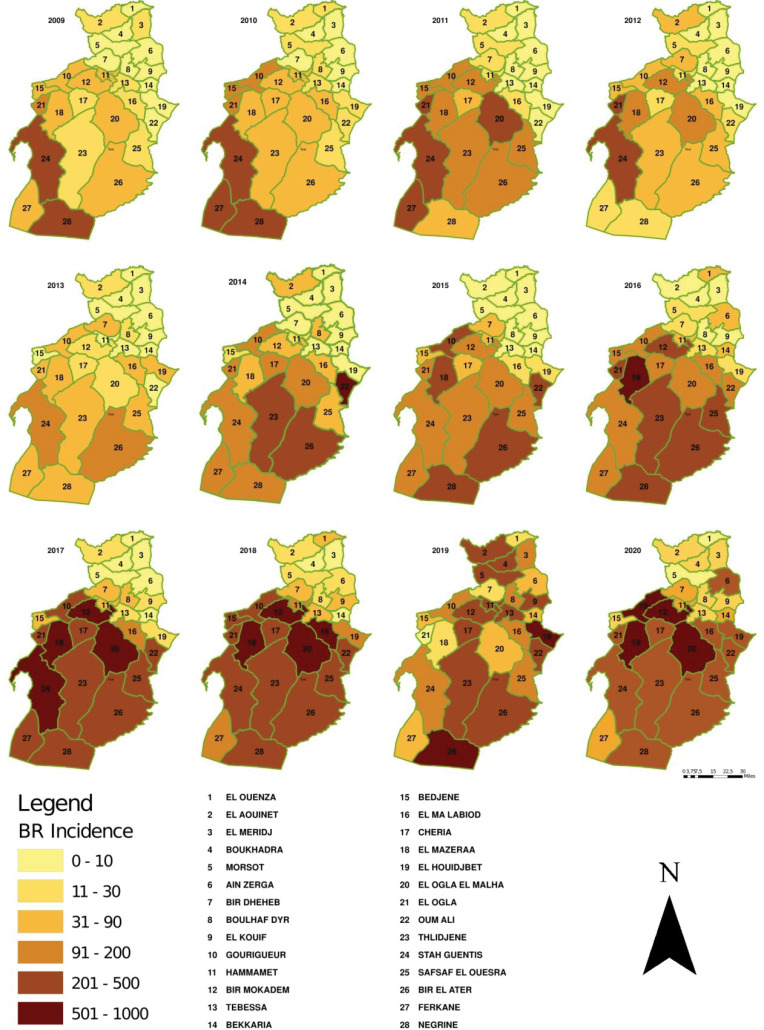
Spatio-temporal distribution of annual human brucellosis incidence by the municipality in Tebessa province from 2009-2020.

 Brucellosis cases were notified throughout the year with peaks in May, June, and July. The seasonality was manifest (Figure 1D). The annual incidence of the province ranged from 30.9 (2013) to 246.7 (2005) per 100 000 persons. The spatial distribution of the incidence (Figure 2) demonstrates that over the years, the human brucellosis was not evenly distributed across municipalities in the province with spatial and temporal variability. As for 2020, 4 out of the 28 municipalities had an incidence higher than 500 per 100 000 people, namely Elogla El Malha (860.8), Gourigueur (544.3), BirMokadem (601.9), El Mazraa (763.7), and almost half of the cases occurred in Bir El Ater (23.6%), Chéria (14.3%), and Tébessa (8.5%).

###  Seasonal autoregressive integrated moving average model

**Figure 3:**
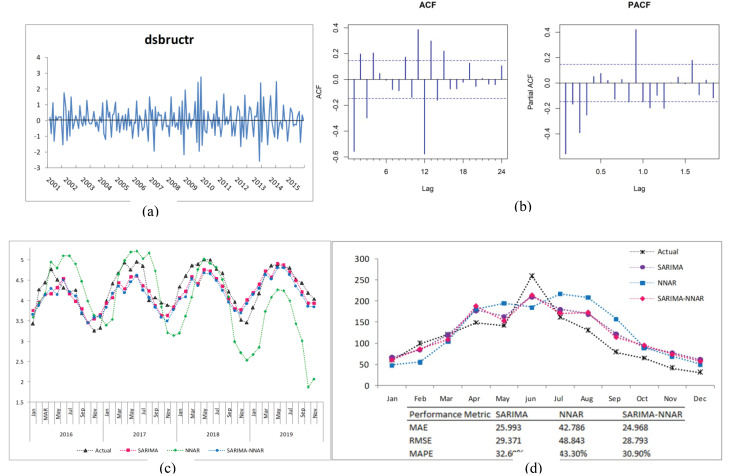
Transformed monthly time series 𝑑𝑠𝑏𝑟𝑢𝑐𝑡𝑟 from 2000-2015 (a), the corresponding autocorrelation function and partial autocorrelation function plots (b), Actual, SARIMA, NNAR, and Hybrid SARIMA-NNAR monthly forecasts of human brucellosis from 2016-2019 (c), as well as Actual, SARIMA, NNAR, and Hybrid SARIMA-NNAR 2020 predictions of human brucellosis with performance metrics (d).

 Prior to model fitting, descriptive statistics of the monthly data were performed, and the time series plot was sketched to evaluate the behavioral pattern in the data (Figure 1C). The monthly reported cases ranged from 2 (December 2008) to 260 (June 2020), and the mean score was obtained at 54.25 ± 48.14 (95% CI: 48.27, 60.21). The training data set, named bruc, exhibits a non-stationary variance, a non-stationary mean, and a seasonal component. To stabilize the variance, the logarithm was applied. To stabilize the mean, a first-order differencing was applied. Peaks occur at lags of 12 months (Figure 3b); therefore, a seasonal differencing was required (𝐷 = 1). The ADF test (𝑡-𝑠𝑡𝑎𝑡𝑖𝑠𝑡𝑖𝑐 = −4.365 and *P* = 0.0005) confirmed the stationary (Figure 3a) of the transformed data time series $dsbructr=\nabla \nabla_{12}\log\left(bruc\right)=\left(1-B\right)\left(1-B^{12}\right)\log\left(bruc\right)$ .

 It is worth noting that 𝑑𝑠𝑏𝑟𝑢𝑐𝑡𝑟 is normally distributed (Jarque-Bera = 2.6734; *P* = 0.2627). It should be mentioned that we lost the first 13 values in the training set; therefore, 179 data points were used in the building process. The autocorrelation and partial autocorrelation functions are displayed in Figure 3b, allowing us to identify possible values for p, q, P, and Q orders. Several possibilities have been considered and on the basis of the best-fitted model criteria (the smallest AIC and RMSE, the highest adjusted $R^{2}$ and the invertibility condition, the significance of AR and MA roots, and the white noise condition for residuals), $SARIMA\left(2,1,3\right){\left(1,1,1\right)}_{12}$ model which fulfills all the features of the best fit model was selected as appropriate (Table 1).

**Table 1:** Parameter estimates of seasonal autoregressive integrated moving average model

	**ar1**	**ar2**	**ma1**	**ma2**	**ma3**	**sar1**	**sma1**
Coefficient	-1.719	-0.903	1.124	-0.109	-0.615	-0.164	-0.721
t-stat	-25.732	-15.677	13.896	-1.049	-8.855	-1.602	-8.620
*P* value	0.001	0.001	0.001	0.293	0.001	0.100	0.001

z_t_=-1.719 z_t-1_-0.903 z_t-2_-0.164 z_t-12_+ $\varepsilon_{t}$ +1.124 $\varepsilon_{t-1}$ -0.109 $\varepsilon_{t-2}$ -0.615 $\varepsilon_{t-3}$ -0.721 $\varepsilon_{t-12}$ z_t_=dsbructr

 The $SARIMA\left(2,1,3\right){\left(1,1,1\right)}_{12}$ was then used to forecast the monthly cases making use of the test set. The simulated values and reported cases matched reasonably well as displayed in Figure 3c with a strong correlation (Pearson product-moment correlation coefficient; 𝑟 = 0.866). Moreover, the monthly predicted values for the year 2020 (Figure 3d) demonstrated a considerable agreement with the actual data with a very strong correlation (𝑟 = 0.915). All these conclusions attest to the adequacy of $SARIMA\left(2,1,3\right){\left(1,1,1\right)}_{12}$ .

###  NNAR and hybrid SARIMA-NNAR models

 A number of 12 time-lagged variables and one hidden layer with six neurons as input features were created while developing the NNAR. Therefore, 180 values were compared in the training set. The $NNAR{\left(12,1,6\right)}_{12}$ with 12 lagged inputs was selected as the best fitting model. The fitted values were highly correlated to actual values (𝑟 = 0.99); nonetheless, the correlation between forecasted and actual values was moderate (𝑟 = 0.539). In the development of hybrid SARIMA-NNAR, the obtained residuals from the SARIMA model were modeled using the NNAR model. The $SARIMA\left(2,0,2\right){\left(1,1,0\right)}_{12}-NNAR{\left(5,1,4\right)}_{12}$ was selected as the best-fitting model. The forecasted and actual values matched very well as depicted in Figure 3c with a very strong correlation (𝑟 = 0.87). Moreover, the predicted and actual values for 2020 were strongly correlated (𝑟 = 0.912).

###  Comparison of the three models

 For the comparison of the fitting accuracy of the three models, the training set was used taking into account lost values for each built model. In fact, for the SARIMA model, the first 13 values in the training set were lost since a first-order difference and a seasonal difference were applied to the original time series to achieve the stationary. The remaining 179 values were used to build the model and then used to compare the fitting accuracy. In a similar vein, for the NNAR model, 180 values were compared for fitting accuracy since 12 time-lagged variables were created as input features, and 179 values were compared for the fitting accuracy of the SARIMA-NNAR model. The test data set was used to compare the forecast performance for the three models. All error measurements of the NNAR model were far lower than those of SARIMA and SARIMA-NNAR models in the training set, and there were slight differences between the metric measures for SARIMA and SARIMA-NNAR models.

 The lowest metric measure values using the test set were achieved by the SARIMA model, followed by the hybrid SARIMA-NNAR model (Table 2). It was concluded that both SARIMA and SARIMA-NNAR models can successfully predict human brucellosis cases. The predictions of cases for 2020 (Figure 3d) indicate that both SARIMA-NNAR and SARIMA models are much better in performance accuracy than the NNAR model and the SARIMA-NNAR model outperforms the SARIMA model. The RMSE, MAE, and MAPE measures for the SARIMA model were far lower than those of the NNAR model, and they were slightly different from those of the SARIMA-NNAR model. The model NNAR gives worse predictions than either SARIMA or SARIMA-NNAR models.

**Table 2:** Comparison of the fitting and forecasting accuracy of the 3 models

**Model**	**Training set**			**Test set**		
	**RMSE**	**MAE**	**MAPE**	**RMSE**	**MAE**	**MAPE**
SARIMA	0.476	0.361	12.488	0.286	0.247	5.962
NNAR	0.131	0.089	3.047	0.808	0.637	19.737
SARIMA-NNAR	0.131	0.250	8.365	0.314	0.272	6.644

RMSE: root mean square error, MAE: mean absolute error, MAPE: mean absolute percentage error.

## Discussion

 Brucellosis was first reported in goats by Cochez (1895) in Algeria in the Oran region.^18^ Currently, no province is spared by this disease which continues to circulate enzootically in different animal populations (e.g., bovine, ovine, caprine, and camelina) with periodic epidemic outbreaks in some provinces. Although the disease benefits from both human and veterinary monitoring programs, limiting the screening of the animal population to dairy cattle herds only on an episodic basis is hampering its eradication.^6^

 The incidence of this disease varies from less than 0.03 to more than 200 per 100 000 people in endemic regions^5^. In Algeria, which is regarded as an endemic area, the mean incidence was reported to be 17.9 over the decade 2008-2017 (95% CI: 14.43, 21.29) and ranged from 10.3 (2013) to 28.4 (2010) per 100 000 population. In Iran, considered also an endemic area, the incidence for almost the same period (2009-2017) ranged from 12.07 (2009) to 25.89 (2014) per 100 000 people. Nevertheless, in Iran, an increasing trend was observed until 2014, followed by a decreasing trend, while in Algeria, a sharp decrease was observed from 2010- 2013, followed by an upward trend.^5,7^

 The current study aimed to assess the epidemiological profile, spatial distribution, and seasonal pattern of human brucellosis, and then develop a model that could accurately predict subsequent brucellosis cases in Tebessa province, an endemo-epidemic region in Algeria.

 Although people can be infected at any age, notifications were significantly higher in people aged 20-65 years. This has also been observed in mainland China.^19^ The male-to-female ratio was in favor of men; studies in Iran, Bosnia, Herzegovina, and Macedonia have shown almost the same ratio.^4,20,21^ The notifications gradually increase from January to April to reach their maximum in May, June, and July and then gradually decrease. A similar seasonal pattern has been observed in other countries as well.^4,22-24^ The trend on notifications throughout the year is partly attributable to the mating period of sheep and goats, which corresponds to late winter and early spring, followed by fetal abortions of infected animals and therefore a period of high risk of infection among people who are in direct or indirect contact with infected animals.

 In recent years, several mathematical models have been developed for brucellosis.^13,19,24,25^ Among the statistical models widely used by researchers, we can refer to SARIMA models since they are considered relevant in seasonal time series prediction. Moreover, the factors that have an impact on the incidence may be omitted with the use of these models. On the other hand, machine learning models, such as ANN, NNAR, and Hybrid SARIMA-NNAR can be appropriate models to approximate various non-linearities in the data and can provide better predictions.^12,13,25-27^ To the best of our knowledge, no mathematical studies have been performed on brucellosis using surveillance data from Algeria.

 In the present study, SARIMA, ANN, and Hybrid SARIMA-NNAR models were built using Tebessa’s human brucellosis data. It was pointed out that the NNAR model outperforms SARIMA and hybrid SARIMA-NNAR models in the training test, and NNAR had lower performance measure values. However, the forecasting accuracy of the three models demonstrates that both SARIMA and SARIMA-NNAR models were preferred for short-term predictions. This study supports the conclusion reached by Maleki et al^27^who statedthat ANN is not always better than the traditional statistical models.

 Brucellosis monitoring in Algeria is based on a passive system. Reporting of confirmed cases and the standardization of treatment are the main measures implemented by health authorities. Neither analysis nor interpretation of the data was undertaken to design intervention strategies. This study, in addition to the analysis and interpretation of the available data, pointed to the usefulness of SARIMA and SARIMA-NNAR models to monitor brucellosis cases and provide estimates of future cases. This knowledge is of great help for predicting if an unusual situation is developing. It could therefore assist decision-makers in having clearer ideas about strengthening the prevention and control measures taken in the province; moreover, it facilitates the initiation of appropriate, effective, and rapid response measures. Therefore, the integration of forecasting models into surveillance systems is essential for assisting public health services plan for the future and ensuring a state of preparedness.

 Every study has some limitations which must be addressed in the paper. Indeed, the risk factors affecting the occurrence and transmission of brucellosis in humans and animals need to be determined; moreover, there is a need for the assessment of the role of environmental and climatic effects on the incidence of this disease. Algeria has instituted a bovine brucellosis control program which has not yielded the expected results. It is therefore necessary to reframe the set objectives and actions to be carried out. Collaborative strategies between human and veterinary health sectors would reduce the burden of brucellosis in affected regions. Moreover, a good management can be achieved by active public participation at all levels of planning, decision-making, implementation, monitoring, and evaluation.

## Conclusion

 The results of the present study indicated that brucellosis was more common in males and the most affected age group was 15-44 years. The peaks in brucellosis notifications occurred in May, June, and July. The disease was not evenly distributed across the province with an upward trend in the last years. As evidenced by the obtained results, both SARIMA and hybrid SARIMA-NNAR models were suitable to predict human brucellosis cases with high accuracy. Reasonable predictions, along with mapping the spatial distribution of the incidence, could assist both veterinary and health policymakers in the development of informed, effective, and more targeted policies, as well as timely interventions.

## Acknowledgments

 The authors thank the TDPH for having provided data for carrying out this study.

## Conflict of interests

 The authors declare that they have no conflict of interest.

**Highlights**

- Overall, 13 670 brucellosis cases were notified in Tebessa province from 2000-2020.
- Brucellosis was more common in males and the most affected age group was 15-44 years.
- The incidence rate ranged from 30.9 (2013) to 246.7 (2005) per 100 000 inhabitants.
- The disease was not evenly distributed across the province with spatial and temporal variability.
- Seasonal autoregressive integrated moving average (SARIMA) and SARIMA - neural network autoregressive (NNAR) were appropriate to predict brucellosis with high accuracy.
